# Plasmon Enhancement Reveals Origin of the Dark States of Photoluminescence Intermittency in Quantum Dots

**DOI:** 10.1002/nap2.70018

**Published:** 2026-01-20

**Authors:** Jialu Li, Zhihao Chen, Guofeng Zhang, Bin Li, Changgang Yang, Wenli Guo, Xue Han, Chuang Wang, Zhuang Ying, Jinhui Wang, Ruiyun Chen, Chengbing Qin, Jianyong Hu, Liantuan Xiao, Suotang Jia

**Affiliations:** ^1^ State Key Laboratory of Quantum Optics Technologies and Devices, Institute of Laser Spectroscopy, Collaborative Innovation Center of Extreme Optics Shanxi University Taiyuan China; ^2^ Key Laboratory of Spectral Measurement and Analysis of Shanxi Province, College of Physics and Information Engineering Shanxi Normal University Linfen China

**Keywords:** band‐edge carriers trapping, colloidal quantum dots, dark states, photoluminescence blinking, plasmon enhancement, single quantum dot spectroscopy

## Abstract

Dark states of photoluminescence (PL) intermittency in colloidal quantum dots (QDs) interrupt PL emission and significantly reduce emission intensity, severely hindering QD applications. However, the origin of dark states remains ambiguous due to their extremely low intensity, which impedes the development of effective suppression strategies. In this study, we use plasmonic gold nanoparticles to significantly increase the radiative rate of excitons, and thereby enhancing the dark‐state PL intensity. Calculations of radiative rate scaling based on the dark‐state PL intensity and lifetime reveal that the dark states originate from band‐edge carrier trapping by collectively activated nonradiative multiple recombination centers (MRCs). Transition states that accompany the dark states are frequently observed in PL trajectories, revealing the presence of a positive feedback mechanism for the activation and deactivation of nonradiative MRCs induced by the phonon kick effect. We perform a Monte Carlo simulation to model the dark and transition states and quantify the nonradiative rates involved. Understanding the origin of dark states can contribute to their suppression, optimization of synthesis, and improvement of performance in QD‐based applications.

## 1 Introduction

Photoluminescence (PL) intermittency, also known as “blinking”, refers to random fluctuations in the PL intensity of single colloidal quantum dots (QDs). The occurrence of PL intermittency has been shown to result in the dissipation of exciton energy as heat [1]. Additionally, this phenomenon is typically accompanied by the quantum‐confined Stark effect, spectral diffusion, and delayed emission [2, 3, 4, 5]. These phenomena have been identified as the primary obstacles for colloidal QDs to serve as single‐photon sources [6, 7]. Furthermore, they have been demonstrated to greatly degrade the performance of QD displays, nanolasers, and optoelectronic devices [8, 9]. This has led to significant hindrances to the application of QDs. Therefore, the capacity to suppress the PL intermittency has emerged as a pivotal metric for assessing the quality of QDs [10, 11, 12]. This relies on a profound understanding of the PL intermittency.

In 1996, Nirmal et al. first observed PL intermittency in single QDs and separated the binary PL trajectories into bright and dark states, attributing the generation of the dark states to nonradiative Auger recombination of trions [13]. The following year, Efros and Rosen proposed the classical charging model [14], which attributes trion formation to Auger autoionization or thermally assisted deep trapping of carriers. To date, the Auger mechanism and the charging model are still widely used to explain the occurrence of dark states of single QDs. With the development of core–shell engineering, in the gradient core–shell interface and fully alloyed QDs, the Auger rate has been suppressed to almost the same order of magnitude as the radiative rate of single exciton (X) [15, 16, 17]. As a result, the PL intensity of the trions, which was originally considered to be the dark states, is not close to the background [18]. Consequently, in Park's work, the relatively strong emission of the negative trion (X^−^) was separately classified as the gray state, whereas the dark state was attributed to the positive trion (X^+^) [19]. This is due to the fact that the X^+^, which contains an extra hole, is more susceptible to the Auger process and has a faster Auger rate compared to X^−^, due to the high density of valence band states and the small effective localization radius of the hole. However, it has recently been reported that X^+^ emission is higher than the background intensity and can be distinguished from the background [20]. Consequently, the origin of the dark states remains elusive and requires reconsideration.

In general, the origin of the various intensity states can be revealed by calculating the scaling of the radiative rate, which can be obtained by extracting the intensity and lifetime values of these intensity states from the PL trajectory. However, the PL intensity of the dark states is extremely low, making them difficult to distinguish from background photons and rendering them challenging to analyze. Background photons mainly stem from dark counts in the detectors, unfiltered laser photons, and fluorescence from the substrates, which complicates the analysis of dark‐state photons.

Here, we use plasmonic gold nanoparticles to enhance the dark state emission of single QDs via plasmon‐exciton interaction. The plasmonic gold nanoparticles increase the radiative recombination of the QDs, which competes with the nonradiative recombination. This greatly enhances the emission of excitons, thereby enabling the dark‐state PL intensity to exceed the background. We measure and analyze the dark state emission using single‐dot PL spectroscopy and Monte Carlo simulations. Radiative rate scaling calculations based on dark‐state PL intensities and lifetimes reveal that dark states are not caused by nonradiative Auger recombination of X^+^ but by nonradiative recombination of band‐edge carrier trapping, which is caused by the collective activation of nonradiative multiple recombination centers (MRCs). Additionally, transition states that accompany the dark states are frequently observed in PL trajectories. This observation reveals the presence of a positive feedback mechanism for the activation and deactivation of nonradiative MRCs induced by the phonon kick effect. We model the dark and transition states via Monte Carlo simulations to quantify their nonradiative rates.

## 2 Results and Discussion

**FIGURE 1:**
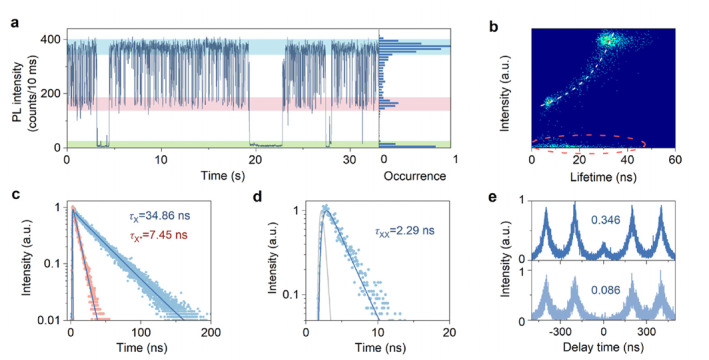
(a) Typical PL trajectory of a single CdSe/ZnS quantum dot (QD) and the corresponding PL intensity histogram. The blue, red, and green highlighted areas from top to bottom indicate the bright, gray, and dark states, respectively. (b) Corresponding fluorescence intensity‐lifetime distribution (FLID) map. The white dashed curve is an inverse proportional function fit, indicating Auger‐blinking. The red elliptical dashed circle indicates the average arrival times of dark‐state photons and their intensities. (c) Corresponding PL decay curves for the bright and gray states, as well as the lifetime values obtained by a single exponential deconvolution fit. The gray lines in the figure represent the instrument response function (IRF) of the system. (d) Corresponding PL decay curve for the biexciton (XX) and the lifetime value obtained by a single exponential deconvolution fit. (e) Corresponding origin (upper panel) and time‐gated (lower panel) second‐order photon correlation function (*g* ^(2)^(τ)) curves.

To investigate the origin of dark states of PL intermittency in colloidal QDs, we synthesized a high‐quality sample of alloyed CdSe/ZnS QDs with a 90% PL quantum yield (QY) via the hot injection method (see Supporting Information S1: Section S2 for more details). Alloyed CdSe/ZnS QDs are representative of Cd‐based QDs used in practical applications and commercialization due to their high PL QY, excellent stability and low band‐edge degeneracy [10, 21, 22, 23, 24]. Figure 1a shows the typical PL trajectory of a single CdSe/ZnS QD with typical PL intermittency behavior. According to the corresponding PL intensity histogram, the PL trajectory can be divided into three states: bright (*I*_b_ = 383 counts/10 ms), gray (*I*_g_ = 165 counts/10 ms), and dark (*I*_d_ = 9 counts/10 ms). The corresponding fluorescence lifetime‐intensity distribution (FLID) map (Figure 1b) is constructed using the method described in the literature [25] in which the lifetime value for each bin is averaged from the arrival times of the PL photons. The PL intensity is inversely proportional to the average lifetime and is fitted by the white dashed line in Figure 1b. This indicates that the observed gray states are due to Auger recombination of trions [26, 27]. The high‐quality CdSe/ZnS alloyed QDs primarily exhibit Auger blinking and long‐duration bright states, attributed to their well‐passivated surfaces that suppress band‐edge carrier trapping (BC) blinking.

The PL decay curves extracted from the bright and gray states are shown in Figure 1c. By performing deconvolution fitting using a monoexponential function, the lifetime values are obtained as 34.86 ns (*τ*_X_) and 7.34 ns (*τ*_X*_). Based on the formula for the scaling of radiative rates *k*_r,X*_/*k*_r,X_ = (*Q*_X*_/*τ*_X*_)/(*Q*_X_/*τ*_X_) = ((*I*_g_ − *I*_bg_)/*τ*_X*_)/((*I*_b_ − *I*_bg_)/*τ*_X_) [28], the scaling of the radiative rates between the gray and bright states is calculated to be 2.01, where *I*_bg_ = 4 counts/10 ms represents the background intensity. Consequently, the bright and gray states in the PL trajectory correspond to the X and trion emissions, respectively. Considering that the QY of bright‐state X is nearly unity [29, 30], the Auger rate of the trion (*k*_A,X*_) is calculated to be 0.0784 ns^−1^ using the formulas *Q*_X*_ = (*I*_g_ − *I*_bg_)/(*I*_b_ − *I*_bg_) = *k*_r,X*_·*τ*_X*_ and *τ*_X*_^−1^ = *k*_r,X*_ + *k*_A,X*_. Note that we cannot distinguish whether this rate corresponds to X^−^ or X^+^ due to the influence of the dielectric screening effect [20].

The Auger rate of another type of trion (X′) with an opposite electric charge can be calculated using the formula *k*_A,XX_ = 2(*k*_A,X*_ + *k*_A,X′_) using the Auger rate of XX (*k*_A,XX_). Therefore, we first need to determine the Auger rate of XX. Figure 1d shows the lifetime of XX as 2.29 ns (*τ*_XX_), obtained by fitting the first photon decay curve of the bright‐state two‐photon events [25]. Subtracting the g^(2)^(0) value of the time‐gated second‐order photon correlation function (g^(2)^(τ)) from the g^(2)^(0) value of the original g^(2)^(τ) in Figure 1e thereby yields the QY of XX (*Q*_XX_) [31, 32], giving *Q*_XX_ = 0.26. Using the scaling formula *k*_r,XX_/*k*_r,X_= (*Q*_XX_/*τ*_X*_)/(*Q*_X_/*τ*_X_), we find that the scaling of the radiative rates between XX and X is calculated to be 3.95, which is consistent with the theoretical value of 4 [19]. This confirms the accuracy of both the XX lifetime and the XX QY measurements. Using the formulas *Q*_XX_ = *k*_r,XX_·*τ*_XX_ and *τ*_XX_^−1^ = *k*_r,XX_ + *k*_A,XX_, we can obtain the Auger rate of XX (*k*_A,XX_) as 0.323 ns^−1^. According to *k*_A,XX_ = 2(*k*_A,X*_ + *k*_A,X′_), the Auger rate of X′ (*k*_A,X′_) is 0.0832 ns^−1^, and the QY of X′ (*Q*_X′_) is 0.41, which is almost as large as the QY of X* (*Q*_X*_) of 0.43 (estimated from the ratio of its intensity to that of the bright state in the PL intensity trajectory in Figure 1a). The similar QYs of the two types of trions may be due to the similar effective localization radius of the electrons and holes in the alloyed QDs [20]. This indicates that they belong to the gray states rather than the dark states. Therefore, X^+^ cannot be considered as the origin of the dark states. The origin of the dark states is not due to the Auger recombination of X^+^ and must be reevaluated. However, the PL intensity of the dark states is not only extremely low but also mixed with the background (Figure 1a). Due to the influence of the background, the average arrival times of the dark‐state photons are scattered in the FLID map (Figure 1b, red elliptical dashed circle). Obviously, the FLID map cannot establish a correlation between dark‐state lifetime and intensity to determine the origin of the dark states. Consequently, more effective methods are required to reveal their origin.

**FIGURE 2:**
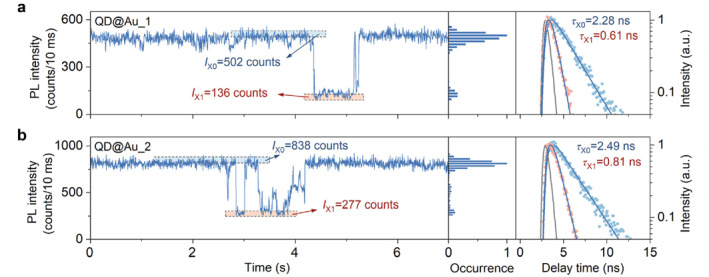
(a, b) Typical PL trajectories of two single QDs with plasmonic gold nanoparticles (QD@Au_1 and QD@Au_2), which exhibit enhanced dark states. The blue and red highlighted areas in dashed boxes indicate the bright and enhanced dark states, respectively. The middle panels show the corresponding PL intensity histograms. The right panels show the corresponding PL decay curves for these states, and the lifetime values were obtained using a single exponential deconvolution fit.

Here, we use plasmonic gold nanoparticles to enhance the dark state emission of single QDs (see more details in Supporting Information S1: Section S2). The plasmonic gold nanoparticles have been demonstrated to enhance the radiative rate of excitons in QDs via the Purcell effect [33, 34, 35]. Figure 2 provides a visual representation of PL intensity trajectories for single QDs under the plasmon‐exciton interaction. The prominent gray states depicted in Figure 1a have now fully disappeared. This phenomenon can be attributed to the competition between ultrafast radiative recombination and nonradiative Auger recombination, which leads to the PL intensity of the trion reaching that of the X (see more details in Supporting Information S1: Section S3) [36, 37, 38]. The PL intensities in the red‐highlighted regions initially in the dark state exhibit a significant increase, far exceeding the background (4 counts/10 ms). This enhancement is representative of most single QDs due to the plasmon‐exciton interaction, although the dark states of some QDs are not enhanced, as will be discussed later. The corresponding PL decay curves of the bright‐state X (highlighted in blue) are shown in the right panels of Figure 2. Due to the plasmon‐exciton interaction, the X lifetime is shortened from about 35 ns to around 2.5 ns and the recombination rate is increased by about 14 times. The increasing radiative recombination process has been shown to significantly enhance the PL intensity of the dark states. The PL decay curves were extracted in the regions highlighted in red (enhanced dark states), and the lifetimes obtained were 0.61 and 0.81 ns, respectively. According to the scaling formula *k*_r,X1_/*k*_r,X0_ = ((*I*_X1_ − *I*_bg_)/*τ*_X1_)/((*I*_X0_ − *I*_bg_)/*τ*_X0_), the radiative rate scalings between the enhanced dark states (red highlighted regions) and the bright states (blue highlighted regions) are 0.96 and 0.99 for QD@Au_1 and QD@Au_2, respectively. Although the ohmic loss of plasmons introduces an additional nonradiative recombination pathway, it does not affect the form of the scaling formula in the plasmon‐exciton coupling system. The scaling values close to 1 indicate that the dark states originate from band‐edge carrier trapping due to the collective activation of nonradiative MRCs [39, 40]. MRCs are typically considered to be composed of QD surface traps that provide nonradiative channels for band‐edge carriers [27, 41, 42]. The rapid collective activation of MRCs significantly increases the nonradiative recombination rates, resulting in dark states.

**FIGURE 3:**
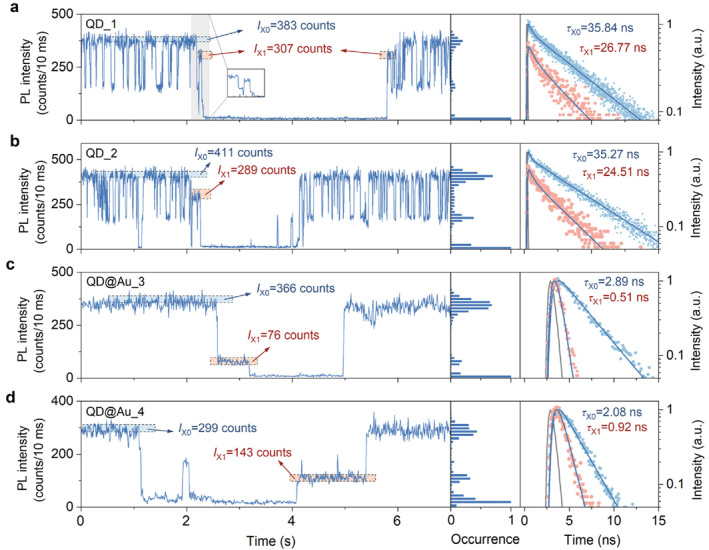
Transition states in the PL trajectories. (a, b) Typical transition states in the PL trajectories for single CdSe/ZnS QDs (QD_1 and QD_2). (c, d) Typical transition states in the PL trajectories for single QDs with plasmonic gold nanoparticles (QD@Au_3 and QD@Au_4). The blue and red highlighted areas in dashed boxes indicate the bright and transition states, respectively. The corresponding PL intensity histograms are shown the middle panels. The corresponding PL decay curves for these states are shown in the right panels. Their lifetime values are obtained by monoexponential deconvolution fitting.

While studying the dark states in the PL trajectories, we often observe intermediate states that accompany them. Figure 3 shows the intermediate states between the bright and dark states (the red highlighted areas), which have uncertain PL intensities and differ from trion emission. These special intermediate states differ fundamentally from the random intermediate states observed in BC‐blinking trajectories. The latter frequently appear in the PL trajectories of single QDs whose surfaces are not well passivated [43]. These special states exist as transition states that occur between the bright states of the Auger‐blinking trajectory and the dark states. They always accompany dark states and appear at the beginning of dark states (Figure 3a,b, red highlights). Based on our research experience [1, 4, 5, 16, 28, 30, 39, 44], this phenomenon is extremely interesting and has never been investigated in depth in previous research. This phenomenon can provide significant insights into the origin of abrupt dark state occurrence.

As shown in Figure 3a, the transition states between the bright and dark states have an intensity of 307 counts/10 ms. The corresponding PL decay curves are shown in the right panel of Figure 3a. Using deconvolution fitting, we obtained the corresponding lifetime of 26.77 ns. By combining the PL intensity and lifetime of the bright state in Figure 3a, the scaling of the radiative rates between the transition state and the bright state is calculated to be 1.07, which is close to 1. This indicates that transition states originate from band‐edge carrier trapping caused by the collective activation of nonradiative MRCs. This origin is the same as that of dark states. The relatively large PL intensities and lifetimes of the transition states suggest that most of the MRCs are inactive for the transition states, with only a few providing nonradiative recombination channels. The nonradiative rates of the transition states are similar in magnitude to the radiative rate of the bright state but much smaller than that of the dark states. Therefore, their PL intensities are much higher than those of the dark states.

In Figure 3b, we observe a similar transition state after 2 s. The scaling of the radiative rates between the transition state and the bright state is calculated to be 1.01, which supports the band‐edge carrier trapping mechanism for the transition state. However, when the dark state returns to the bright state, there is no obvious transition state in Figure 3b. The observation of transition states depends on their duration. A transition state in the PL trajectory can be observed when its duration is longer than the bin time of the PL trajectory. When the duration of the transition state is close to or shorter than the bin time, the transition states become difficult to distinguish from the PL trajectory.

Even though the radiative rates of dark states are enhanced by about 14 times when we use plasmonic gold nanoparticles to enhance the dark state emission of single QDs, a small number of QDs still exhibit dark states with a PL intensity close to the background, as shown in Figure 3c,d. The PL decay curves of these dark states nearly overlap with the instrument response function (see more details in Supporting Information S1: Section S4), which makes it difficult to accurately determine the lifetime of the dark‐state photons. This may be because the nonradiative rates of these dark states are so high that radiative recombination, despite being increased by plasmonic gold nanoparticles, cannot effectively compete with nonradiative recombination. Nevertheless, we found that transition states (highlighted in red) around the dark states are similar to those in Figure 3a,b. We extracted the photons in the transition states and plotted the PL decay curves in the right panels, with the corresponding lifetimes of 0.51 and 0.92 ns, respectively. The scalings of the radiative rates between the transition state and the bright state are 1.05 and 1.04, respectively, indicating that the transition states also originate from band‐edge carrier trapping. Therefore, the transition states accompanying the dark states have the same origin as the dark states. We performed a statistical analysis of the probability of a transition state occurring. Of approximately 50 PL trajectories of single QDs, each of which was observed for several minutes (e.g., Figure S8), roughly half of dark states were accompanied by transition states. In other words, the probability of X transitioning from a bright state to an intermediate state and then to a dark state is about 50%. When transition states were not observed, that is, when X transitions directly from a bright state to a dark state, it may have been due to the fact that the activation time of MRCs was shorter than the sampling time of the PL trajectories. However, these statistical results may not be entirely accurate, as many transition states are difficult to distinguish. Nevertheless, these results suggest that transition states accompanying the emergence of dark states are universal in the high‐quality, alloyed CdSe/ZnS QD samples. Additional examples of transition states are presented in Figures S5, S6, S9 and S10.

Interestingly, the transition states show that once a few MRCs begin to activate, their activation gradually spreads to most of the MRCs. This causes PL trajectories to enter dark states instead of remaining in transition states or returning to bright states. Therefore, we speculate that the activation of nonradiative MRCs involves a positive feedback mechanism. This mechanism is likely induced by the phonon kick effect [45, 46, 47]. When the first band‐edge carrier is trapped by an MRC, the carrier becomes localized. This is followed by vibrational relaxation and the emission of numerous phonons. The emitted phonons then reduce the thermal activation energy of the other MRCs, making them easier to activate and driving continuous carrier trapping and nonradiative recombination. This leads to a rapid increase in the nonradiative rate, ultimately resulting in the formation of dark states.

**FIGURE 4:**
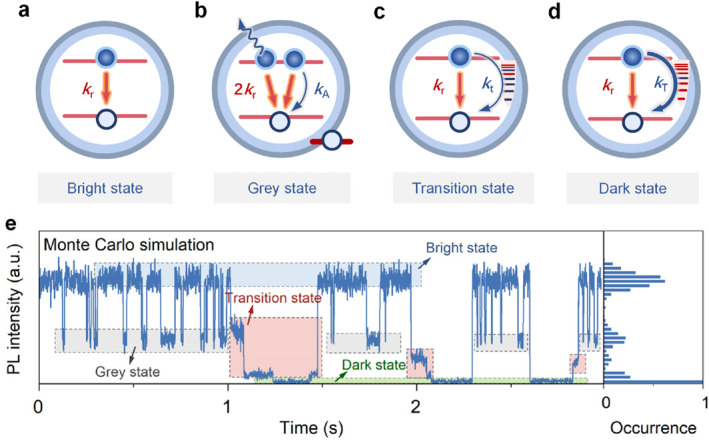
Schematic of the origin of the bright, gray, transition, and dark states, as well as a Monte Carlo simulation of the PL intensity trajectory. (a) Bright state originates from radiative recombination of excitons. (b) Grey state originates from nonradiative Auger recombination of trions. (c) Transition state originates from band‐edge carrier trapping caused by a small number of activated nonradiative multiple recombination centers (MRCs). Activated and inactivated MRCs are indicated by red and blue short lines, respectively. (d) Dark state caused by the activation of more MRCs. The nonradiative trapping rate of the dark state (*k*_T_) is much higher than that of the transition state (*k*_t_). (e) A simulated PL intensity trajectory obtained using the Monte Carlo method. The left panel shows the corresponding PL intensity histogram.

Figure 4 summarizes the origins of the bright, gray, transition, and dark states in single CdSe/ZnS alloyed QDs. The bright state of PL trajectory originates from the radiative recombination of excitons (Figure 4a). Competition between radiative (2*k*_r_) and nonradiative Auger recombination (*k*_A_) of trions leads to the gray state (Figure 4b). The generation of dark states is often accompanied by transition states. These transition states originate from a small number of activated MRCs (Figure 4c). Their nonradiative rate (*k*_t_) is similar in magnitude to the radiative rate (*k*_r_) of X. The existence of transition states reveals that the activation and deactivation of MRCs can occur over a wide range of timescales. The origin of dark states is similar to that of transition states, but more MRCs are activated (Figure 4d). Their nonradiative rates (*k*_T_) are much larger than *k*_r_ and *k*_t_, and their intensity approaches the background.

Figure 4e shows a simulated PL trajectory obtained using the Monte Carlo method to validate the origins of the various intensity states and quantify the nonradiative rates of transition and dark states. The simulated trajectory clearly shows the bright states (blue box), gray states (gray box), transition states (red box), and dark states (green box). The X and trion lifetimes are set to be the same as the QD in Figure 1, and the charging and discharging probabilities follow exponential distributions. This generates the gray state in the trajectory. In accordance with the MRC model, five MRCs with exponentially increasing nonradiative rates were incorporated. The activation and deactivation probabilities of each recombination center also follow an exponential distribution. NRCs with higher nonradiative recombination rates have energy levels farther from the band edge. These deeper energy levels require more energy for activation and deactivation. Thus, these NRCs have lower activation and deactivation probabilities, as well as larger exponential terms. The turn‐on and turn‐off probabilities of band‐edge carrier trapping were set to match the activation and deactivation probabilities of the MRCs with the highest nonradiative rates (see more details in Supporting Information S1: Section S5). This ensures that once activated, all MRCs can participate in the nonradiative recombination channel, driving the PL trajectory from the transition state to the dark state. In the dark state simulation, with all MRCs activated, the total nonradiative recombination rate is 49 times greater than the radiative recombination rate of X, and the PL QY of the dark state is 2%. The dark state in the PL trajectory is almost identical to the background. The positive feedback mechanism is reflected by the fact that activated defect states increase the activation probability of inactive NRCs. Each time an NRC is activated, the exponential terms of the remaining NRCs' activation probabilities with higher nonradiative rates decrease by half of the exponential term of the activated NRC's activation probability. Additionally, due to the limited simulation duration, the occurrence probability of the dark state was significantly increased in the simulated PL trajectories. The simulated PL trajectories exhibit intensity distributions (Figure 4e, right panel) and FLID (Figure S7) that are similar to those of experimental measurements. Thus, the Monte Carlo simulation successfully reproduces the various intensity states in the PL trajectory of single QDs and quantifies the nonradiative rates associated with the transition and dark states.

## 3 Conclusion

We have elucidated the origin of dark states in the PL trajectory of colloidal CdSe/ZnS QDs through plasmon enhancement, single‐dot spectroscopy, and Monte Carlo simulations. Our results show that dark states of PL intermittency originate from band‐edge carrier trapping by collectively activated nonradiative MRCs, rather than nonradiative Auger recombination of trions. The frequent observation of the transition states suggests that the simultaneous activation of MRC involves a positive feedback mechanism related to the phonon kick effect. Once a small number of MRCs begin to activate, the formation of dark states becomes inevitable. Our work reveals the origin of dark states and contributes to optimizing the synthesis of high‐quality QD for applications such as displays, nanolasers, and quantum light sources.

## Author Contributions

**Jialu Li:** conceptualization, methodology, software, data curation, investigation, validation, formal analysis, visualization, project administration, writing – original draft, writing – review and editing. **Zhihao Chen:** methodology, investigation, validation, formal analysis, writing – review and editing. **Guofeng Zhang:** conceptualization, methodology, data curation, investigation, validation, supervision, funding acquisition, project administration, visualization, resources, writing – original draft, writing – review and editing, formal analysis. **Bin Li:** software, methodology, visualization, validation. **Changgang Yang:** software, methodology, validation. **Wenli Guo:** methodology, validation. **Xue Han:** methodology, validation. **Chuang Wang:** methodology. **Zhuang Ying:** software. **Jinhui Wang:** methodology. **Ruiyun Chen:** writing – review and editing, validation, resources. **Chengbing Qin:** validation, writing – review and editing, resources. **Jianyong Hu:** validation, writing – review and editing, resources. **Liantuan Xiao:** supervision, funding acquisition, validation, writing – review and editing, resources, project administration. **Suotang Jia:** funding acquisition, resources.

## Conflicts of Interest

The authors declare no conflicts of interest.

## Supporting information


Supporting Information S1

## Data Availability

The data that support the findings of this study are available from the corresponding author upon reasonable request.
