# Supplementary material for: Plasmon Enhancement Reveals Origin of the Dark States of Photoluminescence Intermittency in Quantum Dots
Source: Nanophotonics. 2026 Jan 20;15(2):e70018. doi: 10.1002/nap2.70018 (PMC12964982; doi:10.1002/nap2.70018)
Supplement: Supplementary file 1 — Supporting Information S1 [file NAP2-15-e70018-s001.docx]

Supporting Material for

Plasmon Enhancement Reveals Origin of the Dark States of Photoluminescence Intermittency in Quantum Dots

Jialu Li,^1,*^ Zhihao Chen,^1,*^ Guofeng Zhang,^1,^^†^ Bin Li,^2^ Changgang Yang,^1^ Wenli Guo,^1^ Xue Han,^1^ Chuang Wang,^1^ Zhuang Ying,^1^ Jinhui Wang,^1^ Ruiyun Chen,^1^ Chengbing Qin,^1^ Jianyong Hu,^1^ Liantuan Xiao, ^1,‡^ and Suotang Jia^1^

^1^ *State Key Laboratory of Quantum Optics Technologies and Devices, Institute of Laser Spectroscopy, Collaborative Innovation Center of Extreme Optics,* *Shanxi University,* *Taiyuan, 030006, China*

^2^ *Key Laboratory of Spectral Measurement and Analysis of Shanxi Province, College of Physics and Information Engineering, Shanxi Normal University, Linfen, 041004, China*

* These authors contributed equally to this work.

†Corresponding author.

guofeng.zhang@sxu.edu.cn (G. Z.)

‡Corresponding author.

xlt@sxu.edu.cn (L. X.)

1. **Experimental setup**

A confocal microscope was used for single-dot PL measurements. The single QDs were excited using a supercontinuum laser (YSL Photonics, SC-Pro) with an output pulse width of ~100 ps and a repetition rate of 5 MHz. The center wavelength is 532 nm and the spectral width is 10 nm. An oil immersion objective with an N.A. of 1.3 was used to collect the PL of single QDs. Finally, two single-photon detectors (Excelitas, SPCM-AQR-15) detected the PL photons according to the HBT scheme. A time-tagged time-resolved time-correlated single-photon counting (TTTR-TCSPC) module recorded the arrival time of each detected photon.

1. **Sample preparation**

The alloyed CdSe/ZnS core/shell quantum dots (QDs) with a gradient chemical composition were synthesized using the “hot injection” method.[1], [2] Figure S1a shows the TEM image of QDs, which exhibit a spherical shape with a diameter of ~13 nm. Figure S1b shows that the photoluminescence (PL) emission peak of the QDs is 625 nm.

The gold nanoparticles (Au NPs), which have a diameter of ~120 nm, were purchased from Beijing Biotech Biotechnology Co., Ltd. Figure S1c shows the experimental and theoretical extinction spectra of the Au NPs in water. The localized surface plasmon resonance (LSPR) peak in water is red-shifted compared to in air. The two sets of extinction spectra demonstrate that the experimental and simulated LSPR peaks are in good agreement, indicating the uniformity of the Au NPs. The simulated extinction spectrum was obtained using the commercial DGTD method package, Lumerical.

Clean glass coverslips (Thermo Scientific, 18 mm × 18 mm) were immersed in a 1% aqueous solution of 3-aminopropyltrimethoxysilane for 10 minutes to silanize their surface. The Au NPs solution was dropped onto the silanized glass coverslips and allowed to dry slowly. Al_2_O_3_ films with a thickness of 2.5 nm were deposited on a glass substrate with Au NPs using atomic layer deposition (ALD) to prevent charge transfer from quenching the PL of the QDs. The AFM image of the substrate is shown in Figure S1d. Finally, a QD solution with a concentration of 10^-9^ mol/L was spin-coated onto the substrate. The QDs are much smaller than the Au NPs, and the dispersion of the QDs on the coverslip is much lower than that of the Au NPs. This ensures the preparation of a coupled system with one Au NP and one QD.


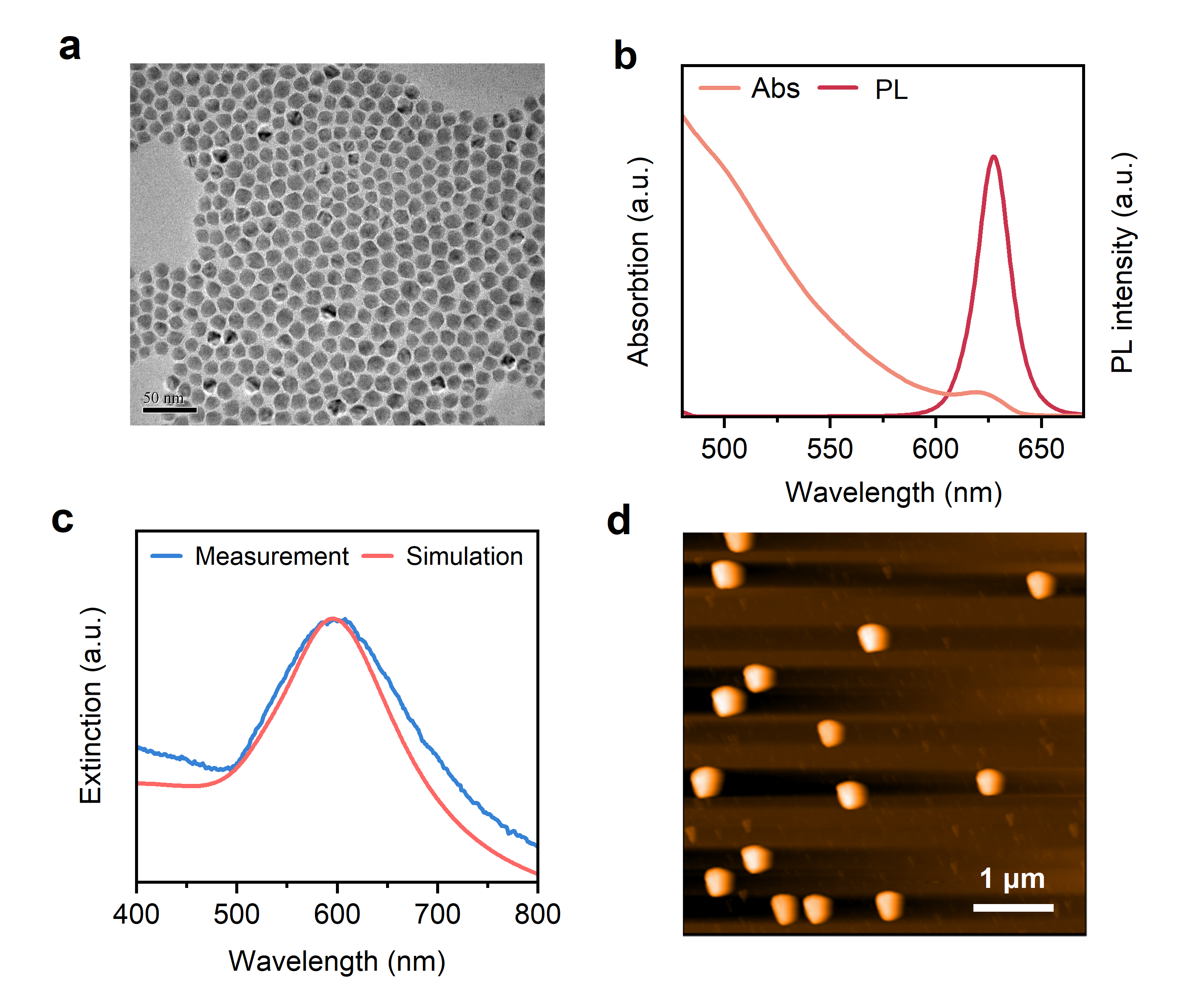


**Figure S1.** (a) Transmission electron microscope (TEM) images of alloyed CdSe/ZnS QDs. (b) Absorption and photoluminescence (PL) spectra of alloyed CdSe/ZnS QDs dispersed in toluene. (c) Experimental (blue) and theoretical (red) extinction spectra of 120 nm Au NPs in water. (d) Atomic force microscope (AFM) images of Au NPs dispersed on a silanized coverslip.

1. **Time-gating-intensity trajectory method for extracting lifetime of trion in single QD@Au**

The time-gating-trajectory method involves subtracting the portion of the photoluminescence (PL) trajectory in which the average photon arrival time exceeds the time gate. In the time-gated PL intensity trajectory, the PL intensity of the short-lifetime exciton decreases significantly, while the PL intensity of the X decreases less. This allows for the separation of exciton states with different lifetimes that were originally mixed in the bright state. Figure S2 shows the time-gated intensity trajectories (red curves) of QD@Au_1 and QD@Au_2. Compared to the original trajectories, the short-lifetime exciton components are separated from X. We plotted the PL decay curves (red dots) of the short-lifetime exciton in the red-highlighted regions in the right panel. The corresponding lifetimes were fitted as 1.07 ns and 1.39 ns, respectively. Considering that the relative quantum yields (QY) of the short-lifetime excitons and X in the original trajectories are close to unity, the scaling of the radiative rates between the short-lifetime excitons and X is found to be 2.13 and 1.93, respectively, according to the formula *k*_r,X*_/ *k*_r,X_=(*Q*_X*_/*τ*_X*_)/(*Q*_X_/*τ*_X_). This proves that the short-lifetime exciton is trions, and their PL intensity is nearly equal to that of X.


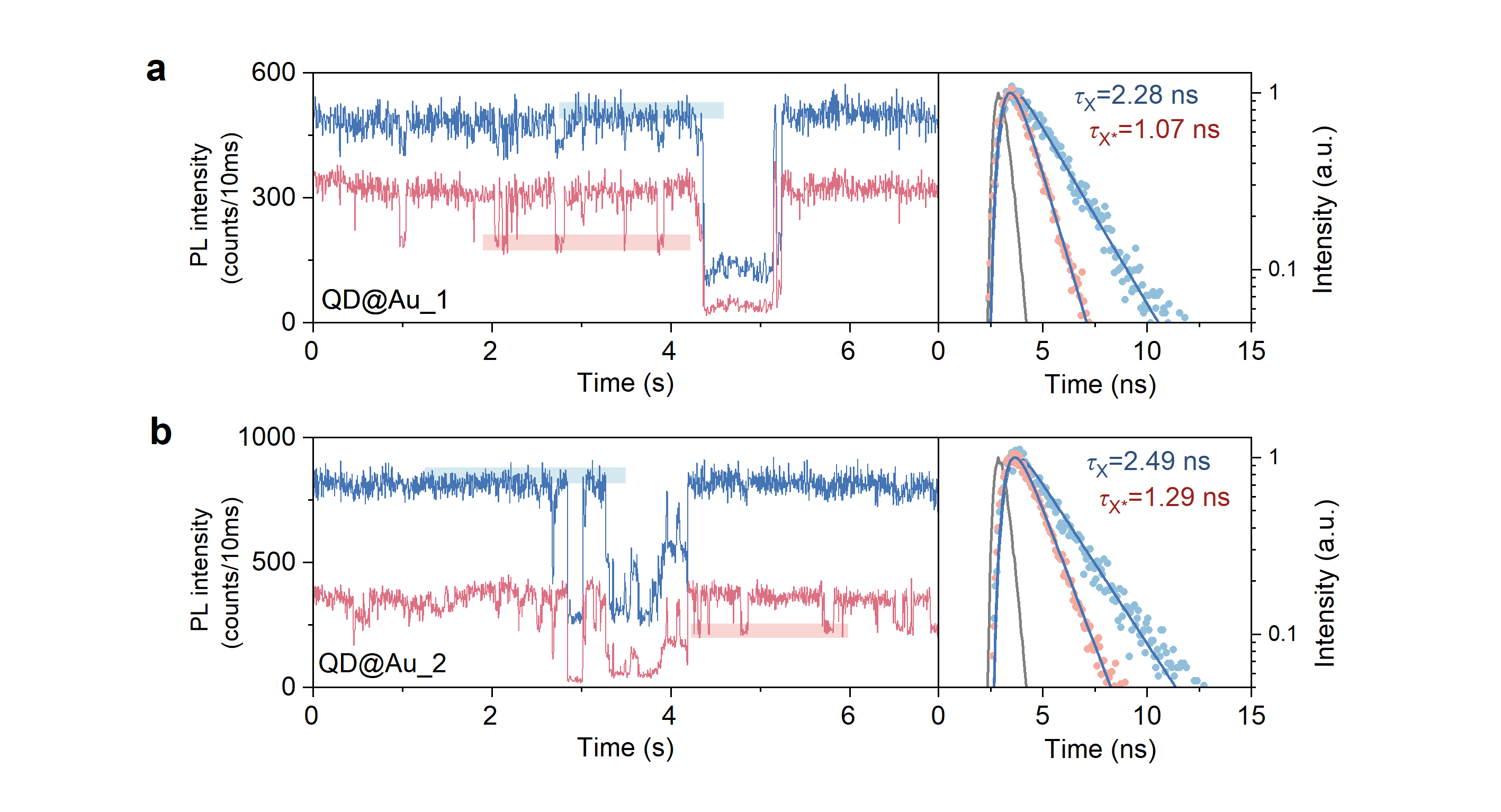


**Figure S2.** (a) Original (blue) and time-gated (red) PL trajectories of QD@Au_1 in Figure 3a. The time gate is set at 4 ns. The PL decay curves of X (blue highlighted area) and the short-lifetime exciton (red highlighted area) are plotted in the right panel. (b) The original (blue) and time-gated (red) PL trajectories of QD@Au_2 in Figure 3b. The time gate is set at 5 ns. The PL decay curves of X (blue highlighted area) and short-lifetime exciton (red highlighted area) are plotted in the right panel. All decay curves are fitted using monoexponential deconvolution. The gray curve represents the instrument response function.

1. **PL decay curves of dark states in single QD@Au**

We extracted the dark-state photons (green highlight) from the PL trajectories of QD@Au_3 and QD@Au_4, and plotted the PL decay curves (green dots) in Figure S3. As can be seen, the PL decay curves are close to the instrument response functions (grey lines), making it impossible to accurately fit lifetime values.


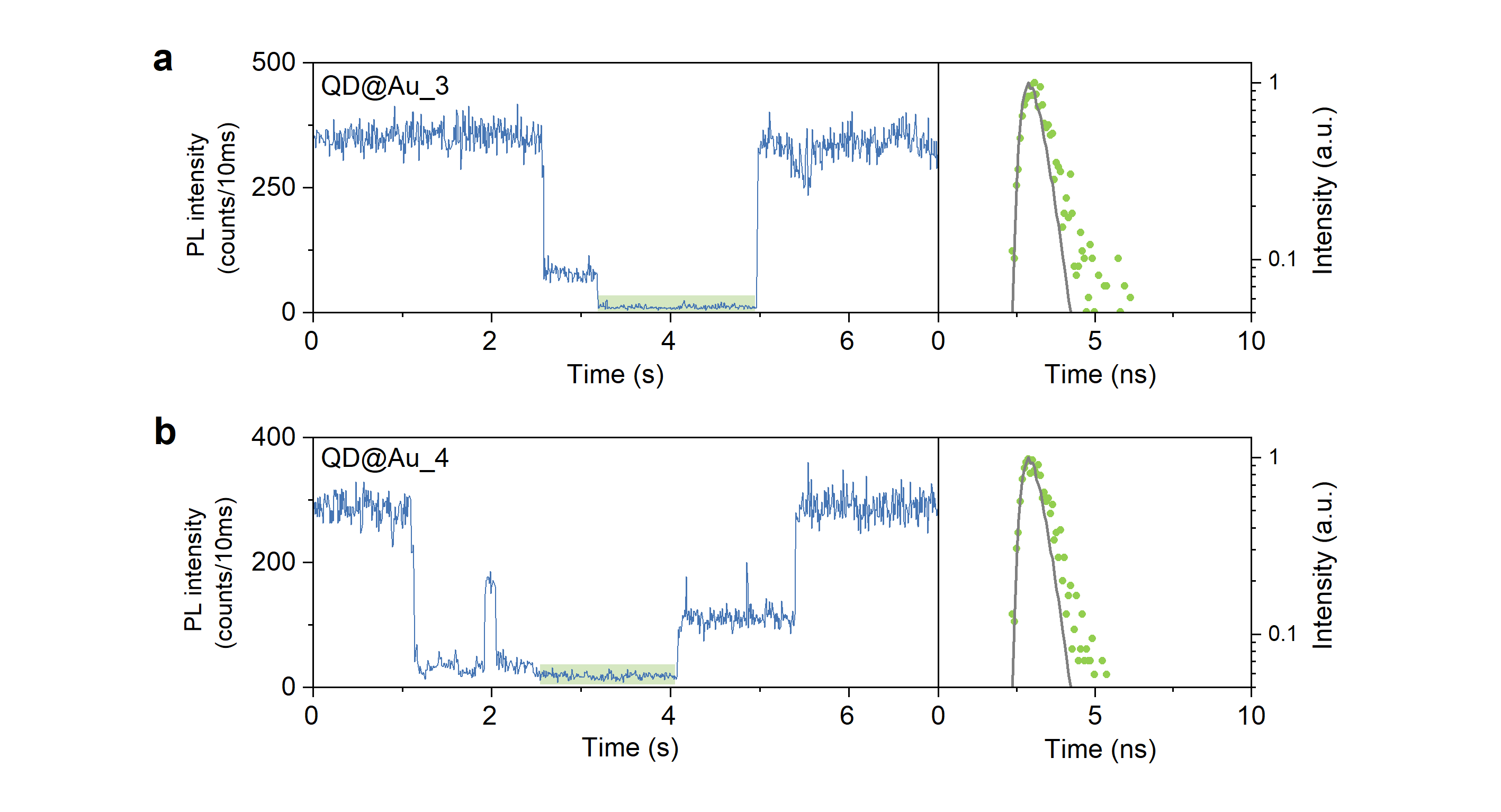


**Figure S3.** (a, b) The PL trajectories (blue) of QD@Au_3 in Figure 3c and QD@Au_4 in Figure 4c, as well as the PL decay curves of the dark-state photons in the green highlighted regions, are plotted in the right panel. The gray curve represent the instrument response function.

1. **Parameter Setting in Monte Carlo Simulations**

During the simulation, we neglected the collection efficiency of the confocal system, and the excitation parameters were consistent with those of the experiment. The bin time of the PL trajectory was set to 1 ms. The switching of Auger-blinking depends on charging and discharging probabilities that follow exponential distributions. The exponential terms were set to 0.042 s and 0.012 s, respectively. The X and trion lifetimes are the same as those of the QD in Figure 1 as 34.86 ns and 7.45 ns, respectively. The QY of the trion is 0.43.

The nonradiative rates of the five nonradiative recombination centers increase exponentially, with values of 0.007 ns^-1^, 0.024 ns^-1^, 0.086 ns^-1^, 0.294 ns^-1^ and 1.025 ns^-1^, respectively. The total nonradiative rate (1.406 ns^-1^) is 49 times the radiative rate of X. The activation and deactivation probabilities of each recombination center follow an exponential distribution as well. NRCs with higher nonradiative recombination rates have energy levels farther from the band edge. These deeper energy levels require more energy for activation and deactivation, and thus have lower activation and deactivation probabilities, as well as larger exponential terms. The activation probabilities sequentially decrease with exponential terms of 0.03 s, 0.05 s, 0.08 s, 0.2 s, and 0.3 s, while the deactivation probabilities’ exponential terms are 0.02 s, 0.025 s, 0.036 s, 0.2 s, and 0.315 s. The positive feedback mechanism is reflected by the fact that activated defect states increase the activation probability of inactive NRCs. Each time an NRC is activated, the exponential terms of the remaining NRCs’ activation probabilities with higher nonradiative rates decrease by half the exponential term of the activated NRCs’ activation probability. Additionally, due to the limited simulation duration, the occurrence probability of the dark state was significantly accelerated in the simulated PL trajectories. The switching of band-edge carrier trapping (BC) blinking depends on the same exponential terms as the activation and deactivation probabilities of the recombination center with the highest nonradiative rates as 0.3 s and 0.315 s, respectively. Due to the randomness of NRC activation, it is impossible to obtain PL trajectories that are completely identical to those of QD_1 and QD_2 in Fig. 3, as well as QD_3 to QD_12 in Fig. S5 through simulations.


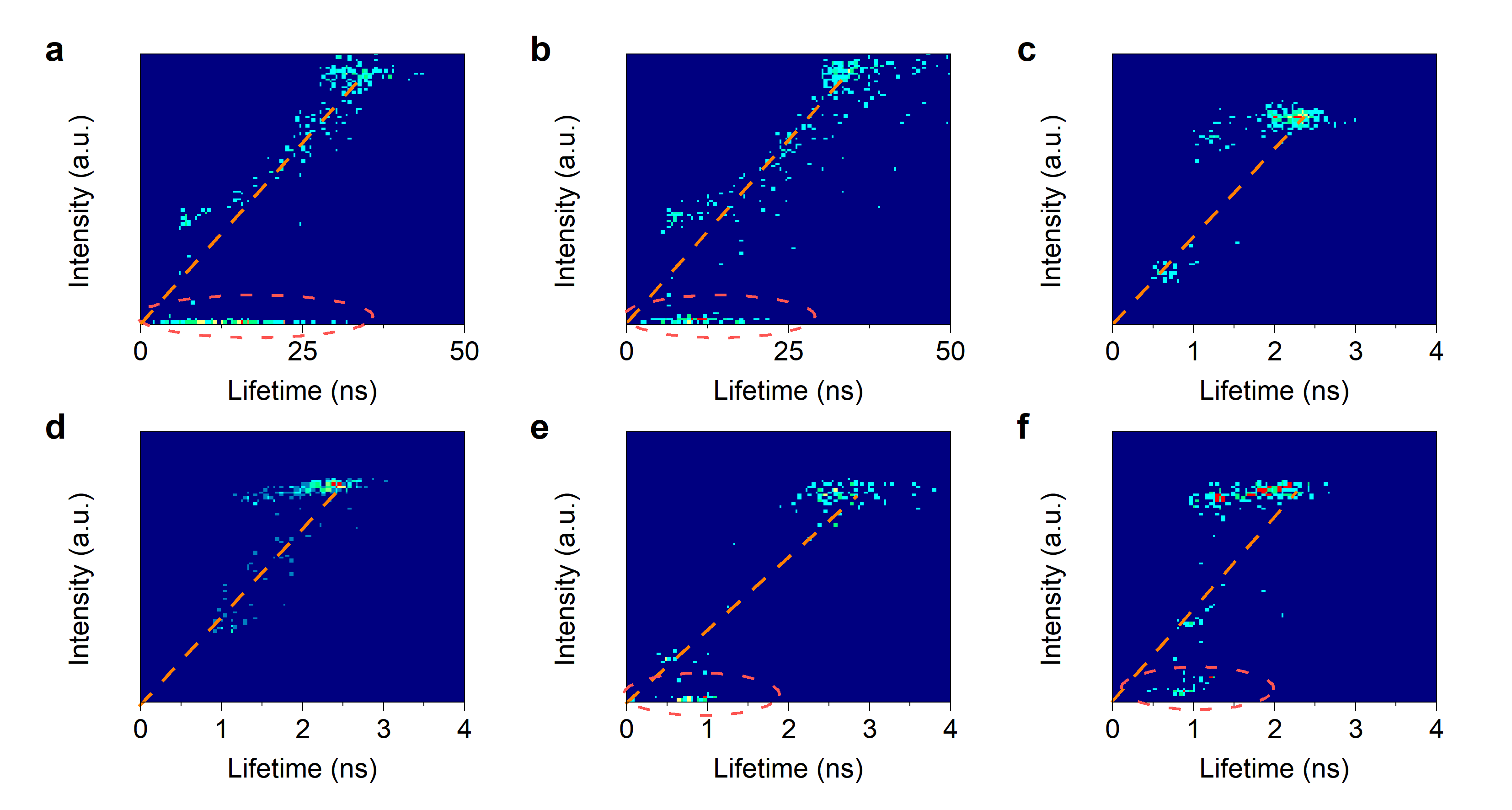


**Figure S4.** (a-f) Corresponding fluorescence intensity-lifetime distribution (FLID) maps of QD_1, QD_2, QD@Au_1, QD@Au_2, QD@Au_3, and QD@Au_4, respectively. The orange dashed lines indicate band-edge carrier trapping (BC) blinking. The red elliptical dashed circles indicate the average arrival times of dark-state photons and their intensities.


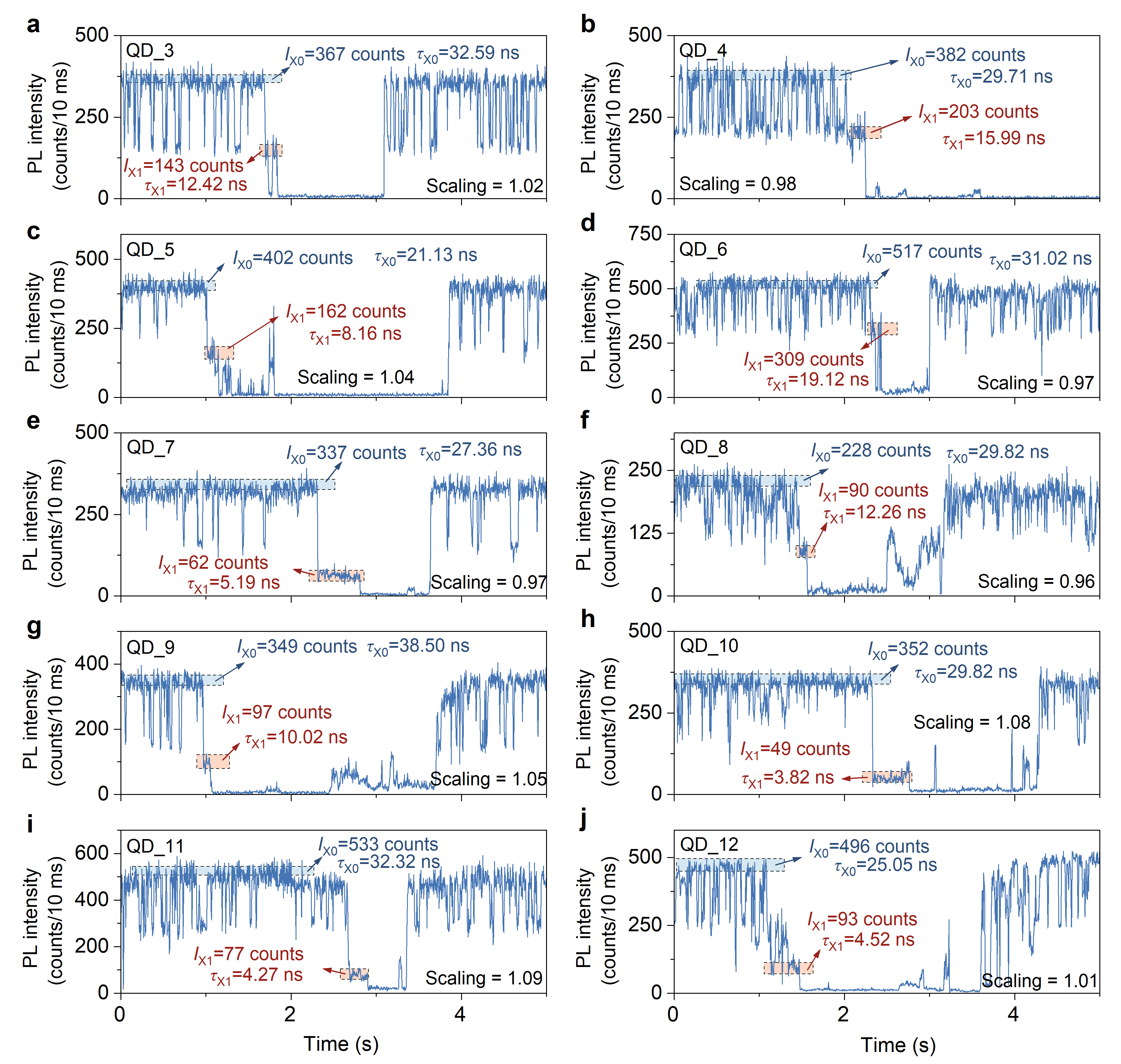


**Figure S5.** (a-j) Additional examples of transition states in PL trajectories of single QDs. The blue and red highlighted areas indicate the bright and transition states, respectively. The lifetime values were obtained by fitting the decay curves of photons in the dashed boxes using a single exponential function.


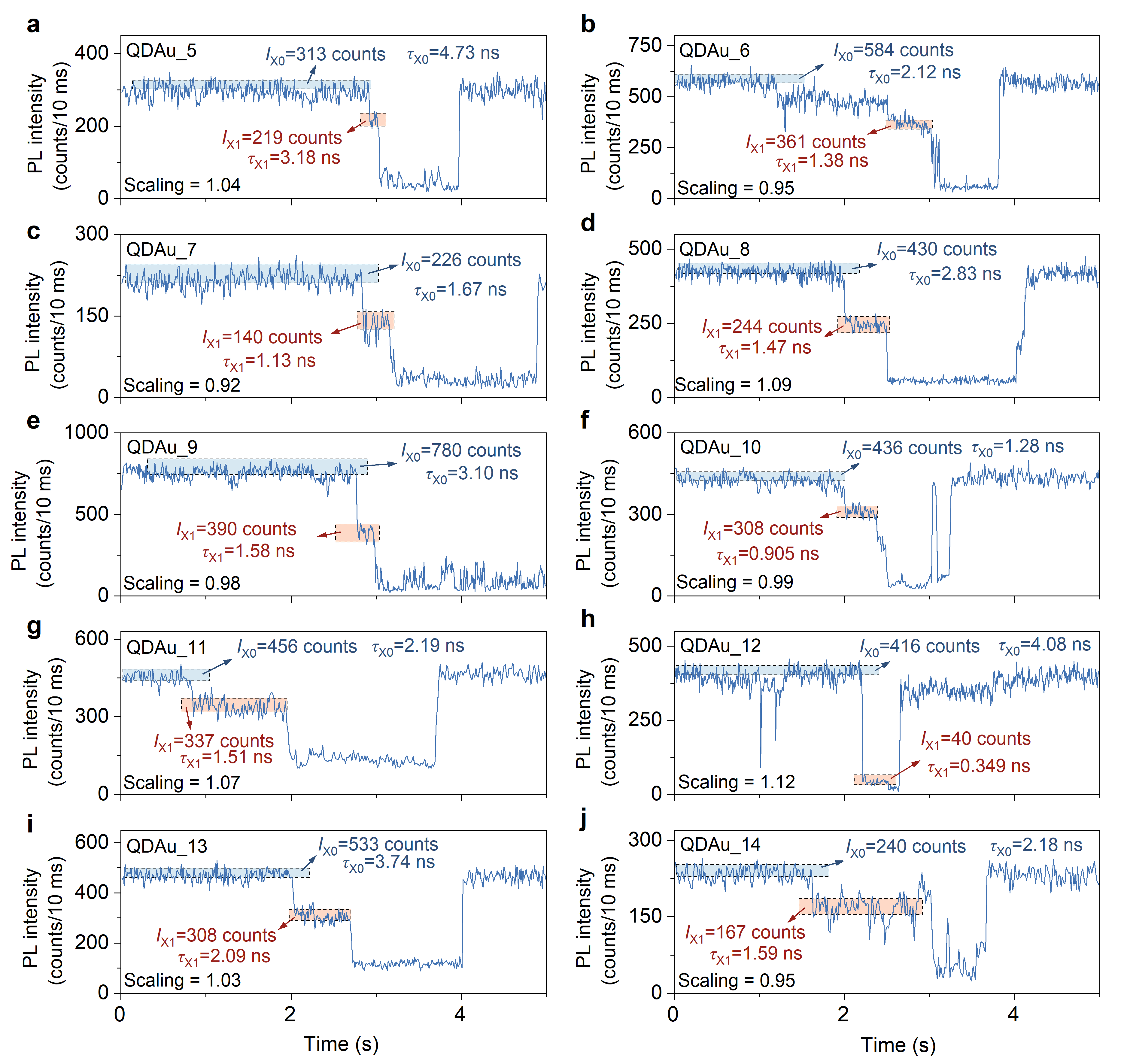


**Figure S6.** (a-j) Additional examples of transition states in PL trajectories of single QD@Aus. The blue and red highlighted areas indicate the bright and transition states, respectively. The lifetime values were obtained by fitting the decay curves of photons in the dashed boxes using a single exponential function.


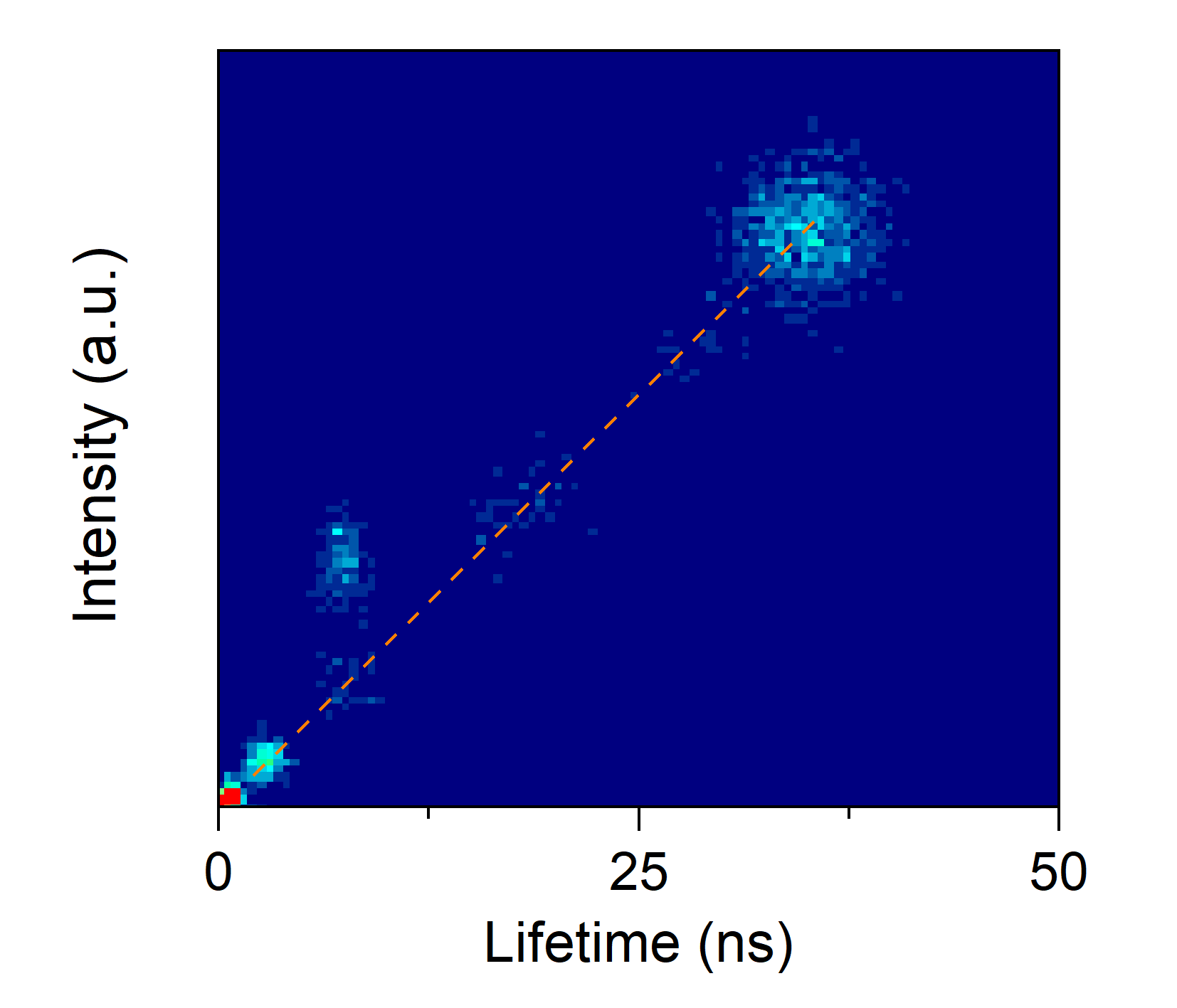


**Figure S7.** Corresponding FLID maps of the simulated PL intensity trajectory in Fig. 4e obtained using the Monte Carlo method. The orange dashed lines indicate BC blinking.


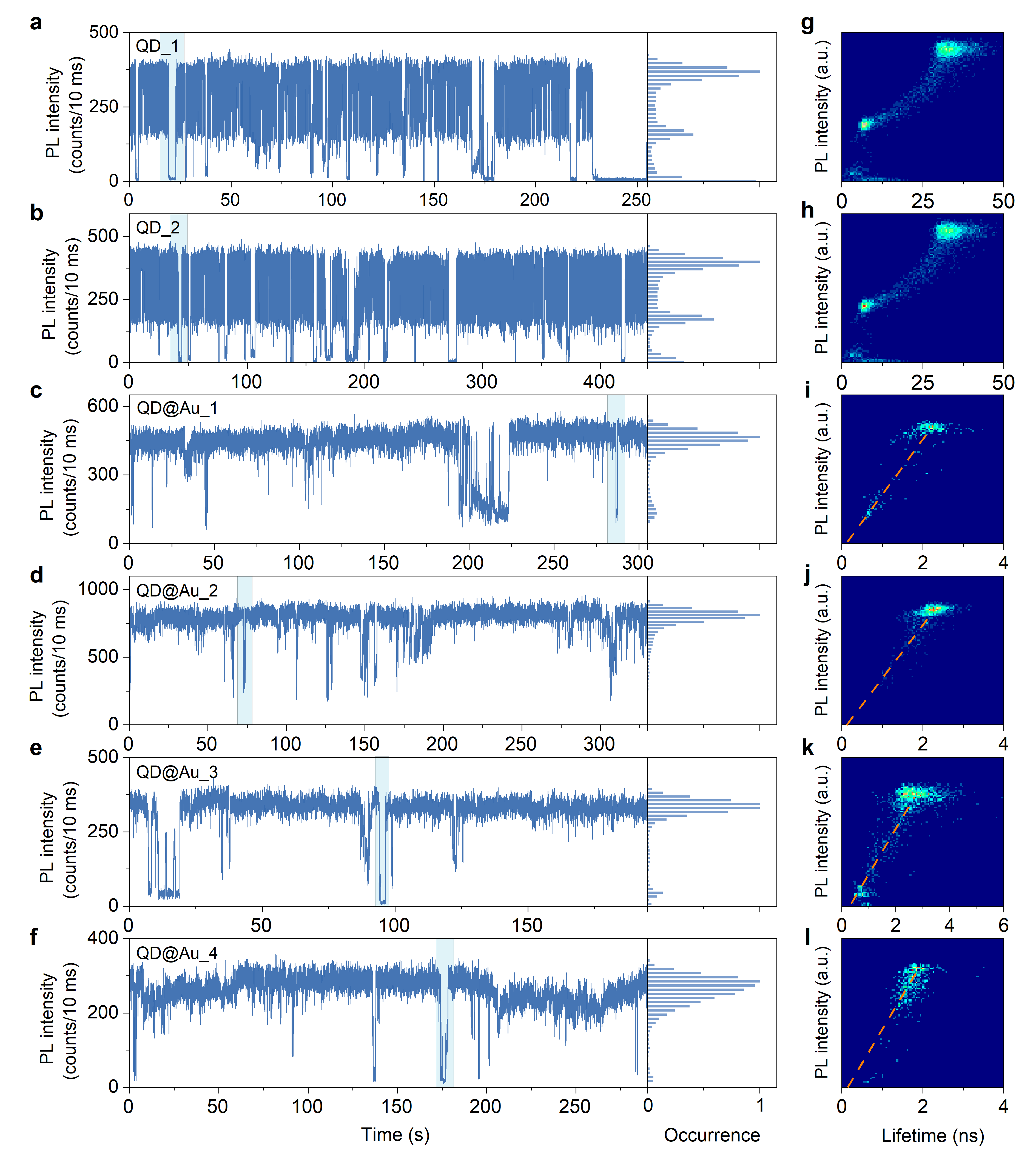


**Figure S8.** (a-f) The complete PL trajectories of QD_1, QD_2, QD@Au_1, QD@Au_2, QD@Au_3, and QD@Au_4 (shown in Figs. 2 and 3 for the blue regions, only). These trajectories cover a time span of several minutes. The corresponding PL intensity histograms are shown in the right panels. (g-l) Corresponding FLID maps of the PL trajectories in (a-f). The orange dashed lines indicate band-edge carrier trapping (BC) blinking.


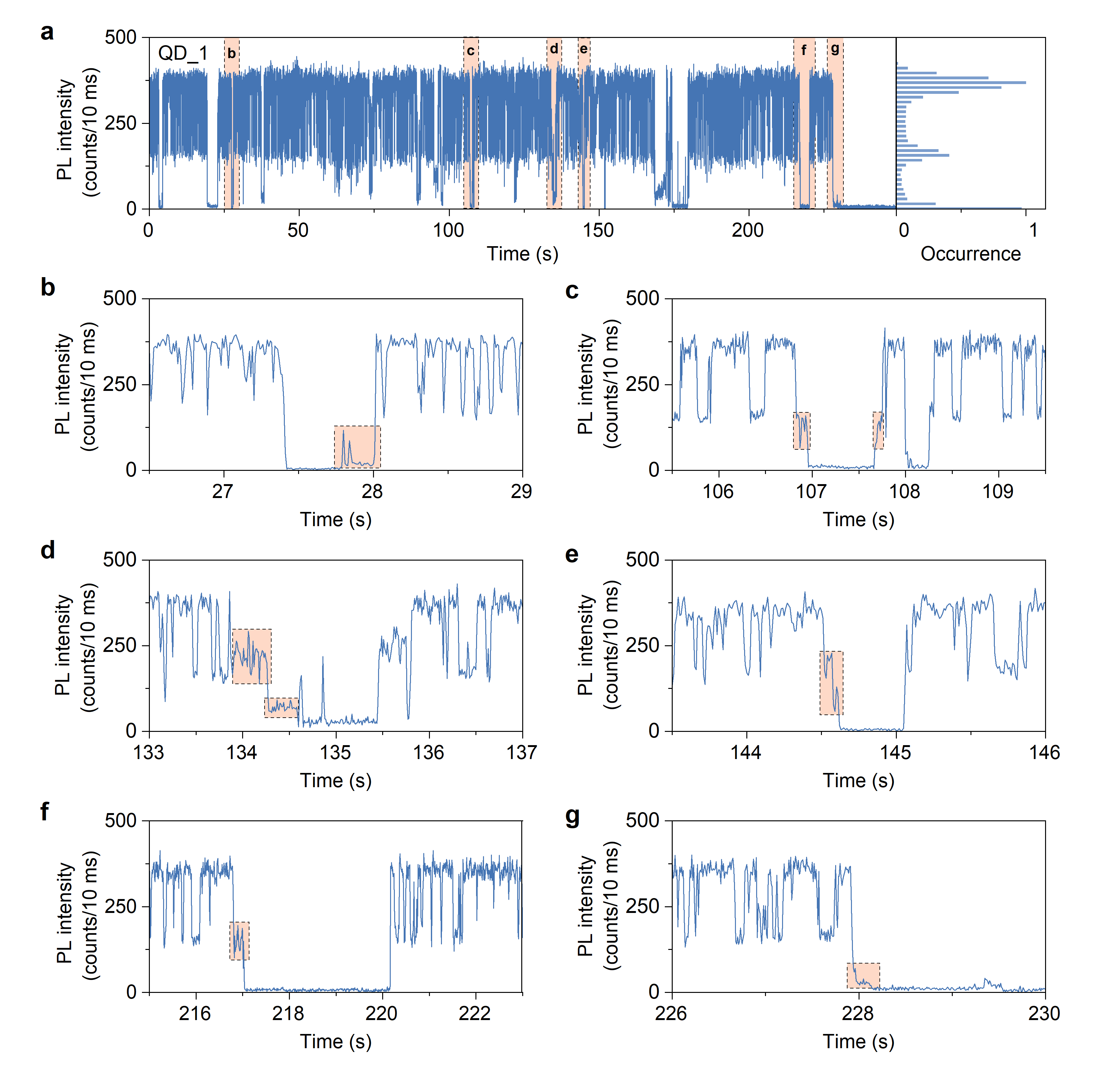


**Figure S9.** (a) The complete PL trajectory of QD_1 in Fig. 3. The corresponding PL intensity histogram is shown in the right panel. (b-g) Enlargements of the red regions in (a). The red regions indicate the typical transition states.


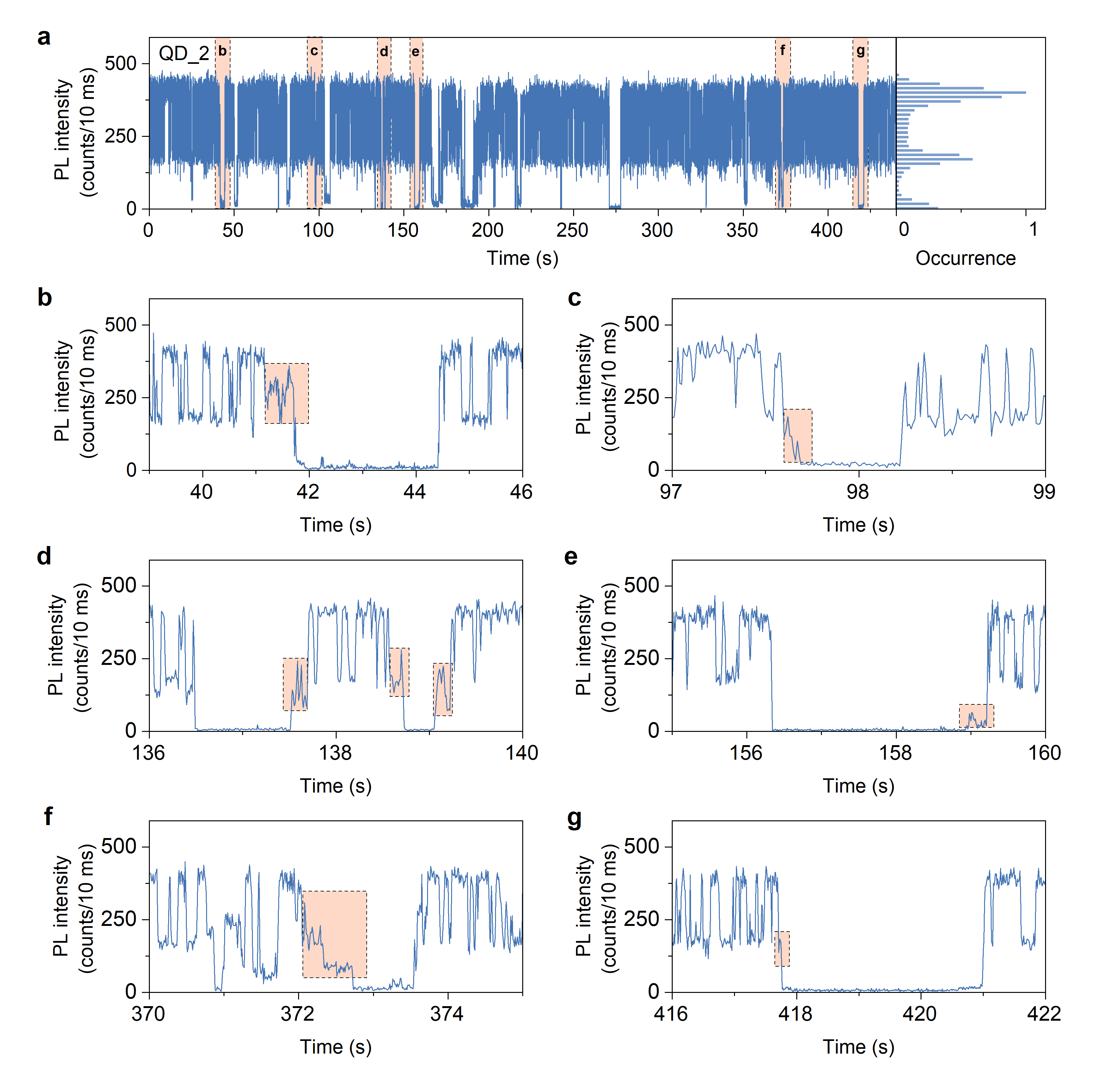


**Figure S10.** (a) The complete PL trajectory of QD_2 in Fig. 3. The corresponding PL intensity histogram is shown in the right panel. (b-g) Enlargements of the red regions in (a). The red regions indicate the typical transition states.

**References**

[1] J. Cho, Y. K. Jung, and J. K. Lee, “Kinetic studies on the formation of various II–VI semiconductor nanocrystals and synthesis of gradient alloy quantum dots emitting in the entire visible range”, *J. Mater. Chem.*, 22, 10827 (2012).

[2] C. Yang *et al.*, “Conversion of photoluminescence blinking types in single colloidal quantum dots”, *Small*, 20, 2309134 (2024).
